# Rethinking *Clostridioides difficile* infection (CDI) surveillance definitions based on changing healthcare utilisation and a more realistic incubation period: reviewing data from a tertiary-referral hospital, Ireland, 2012 to 2021

**DOI:** 10.2807/1560-7917.ES.2024.29.6.2300335

**Published:** 2024-02-08

**Authors:** Mairead Skally, Kathleen Bennett, Hilary Humphreys, Fidelma Fitzpatrick

**Affiliations:** 1 Department of Microbiology, Beaumont Hospital, Dublin, Ireland; 2 Department of Clinical Microbiology, RCSI University of Medicine and Health Sciences, Dublin, Ireland; 3 European Society of Clinical Microbiology and Infectious Diseases (ESCMID) Study Group for Clostridioides difficile – ESGCD, Basel Switzerland; 4 These authors contributed equally to this work and share first/last authorship; 5 Data Science Centre, School of Population Health, RCSI University of Medicine and Health Sciences, Dublin, Ireland

**Keywords:** *Clostridioides difficile* infection, CDI, hospital epidemiology, case definition, CDI surveillance

## Abstract

**Background:**

Community-associated *Clostridioides difficile* infections (CA-CDI) have increased worldwide. Patients with CDI-related symptoms occurring < 48 hours after hospitalisation and no inpatient stay 12 weeks prior are classified as CA-CDI, regardless of hospital day attendances 3 months before CDI onset. Healthcare-associated (HA) CDIs include those with symptom onset ≥ 48 hours post hospitalisation.

**Aim:**

To consider an incubation period more reflective of CDI, and changing healthcare utilisation, we measured how varying surveillance specifications to categorise patients according to their CDI origin resulted in changes in patients’ distribution among CDI origin categories.

**Methods:**

New CDI cases between 2012–2021 from our hospital were reviewed. For patients with CA-CDI, hospital day attendances in the 3 months prior were recorded. CA-CDI patients with hospital day attendances and recently discharged CDI patients (RD-CDI; CDI onset 4–12 weeks after discharge) were combined into a new ‘healthcare-exposure’ category (HE-CDI). Time from hospitalisation to disease onset was varied and the midpoint between optimal and balanced cut-offs was used instead of 48 hours to categorise HA-CDI.

**Results:**

Of 1,047 patients, 801 (76%) were HA-CDI, 205 (20%) CA-CDI and 41 (4%) were RD-CDI. Of the CA-CDI cohort, 45 (22%) met recent HE-CDI criteria and, when reassigned, reduced CA-CDI to 15%. Sensitivity analysis indicated a day 4 cut-off for assigning HA-CDI. Applying this led to 46 HA-CDI reassigned as CA-CDI. Applying both HE and day 4 criteria led to 72% HA-CDI, 20% CA-CDI, and 8% HE-CDI (previously RD-CDI).

**Conclusion:**

CDI surveillance specifications reflecting healthcare exposure and an incubation period more characteristic of *C. difficile* may improve targeted CDI prevention interventions.

**Key public health message**

**What did you want to address in this study?**

Community-associated (CA) *Clostridium difficile* infection (CDI) patients have symptom onset < 48 hours post hospitalisation (regardless of recent prior day attendances at a hospital) and no inpatient stay in the prior 12 weeks. Hospital-associated (HA) CDIs include inpatients with symptom onset ≥ 48 hours post admission. We studied if cut-off time discerning HA and CA-CDI and considering prior hospital day attendances impacted assessment of CDI origin.

**What have we learnt from this study?**

Our analysis identified a day 4 cut-off for healthcare-associated (HA)-CDI (instead of 48 hours). In our patient cohort, applying this cut-off for HA-CDI, and considering prior hospital day attendances in a new ‘health-exposures’ (HE) category, did not hugely skew patient distributions by CDI origin. It did however result in a classification of CDI origin more reflective of *C. difficile’s* incubation period and of patients’ exposure to a healthcare setting.

**What are the implications of your findings for public health?**

To address CDI, information on where infection is acquired is important for decision-making both locally and nationally. A review of CDI categories by origin is needed to include day attendances at hospital and an incubation period more suited to *C. difficile*. This could possibly help to identify transmission reservoirs that might not be detected using current definitions, potentially improving support for infection control and antimicrobial stewardship measures.

## Introduction

Surveillance definitions are the cornerstone of epidemiology and facilitate interrogation of trends, comparison of infection rates within and between countries, and highlight areas for improvement. Those for *Clostridioides difficile* infection (CDI) were first piloted in 2013 in Europe and have been adopted by most national CDI surveillance systems for assessing trends and benchmarking [1,2]. CDI surveillance definitions classify cases according to origin and are used for local, national, and European reporting [3,4]. For cases where the time between admission and onset of symptoms is greater than 48 hours, the classification which dates to the 1970s, is healthcare-associated CDI (HA-CDI) [5]. This includes patients re-admitted with CDI symptoms within 4 weeks of discharge, as well as patients, who are resident in long-term care facilities. Community-associated CDI (CA-CDI) are those patients who have symptoms in the community prior to admission or within 48 hours of hospitalisation and have had no inpatient stay in the previous 12 weeks. The ‘recently discharged’ origin, i.e. RD-CDI (also referred to as ‘unknown’ or ‘indeterminate’) is applied if a patient has been discharged in the previous 4–12 weeks [3,4,6].

In 2021 an international increase in CA-CDI was reported impacting younger people (median age of 50 vs 72 years for HA-CDI) with a lower mortality than HA-CDI, and with a range of *C. difficile* community reservoirs proposed, including humans, animals, food sources and the environment [7]. There is evidence that the difference between HA-CDI and CA-CDI strains is less marked, with one European point prevalence study over 6 months in 2018 with 118 sites in 12 countries participating, reporting 26 *C. difficile* ribotypes (RTs) across both HA-CDI and CA-CDI representing 68.9% (193/280) of isolates [8]. Likewise, a Canadian prospective study suggested that the strains from 78 patients with CA-CDI collected in 2012 were indistinguishable from HA-CDI [9].

The reasons for the similarities between HA-CDI and CA-CDI strains remain unclear, however, it is thought that environmental sources such as food, water, and animals as well as colonised patients post discharge from healthcare facilities, may play an important role in *C. difficile* spore dispersal, increasing exposure of people to *C. difficile* outside of the healthcare setting [10]. We recently reported that, in our institution, we have observed an increase in CA-CDI in hospitalised patients and a similarity of dominant RTs between HA-CDI and CA-CDI [11]. In addition, we also observed that patients are increasingly utilising healthcare day services such as day surgery, day wards (e.g. oncology and renal dialysis) in our hospital with outpatient activity increasing by 21% over the period between 2012 and 2021 [11]. These attendances do not require overnight hospital admission but still result in patient exposure to healthcare services, professionals/practitioners and the hospital environment. It could be argued that CDI patients, with such attendances, who live in their own homes, and are not admitted prior to CDI onset, are a separate category in terms of perspectives of CDI risk, infection prevention and control (IPC) and antimicrobial stewardship. Current CDI case definitions mean that these patients, who have some healthcare exposure, are categorised as CA-CDI. This may lead to a possible overestimation of CA-CDI. As these patients are counted as CA-CDI, they are in fact not being actively monitored or trended over time. In our hospital our current CDI prevention programmes are focused primarily on interventions for hospitalised patients. There is less focus on patients who attend the hospital for day services and these patients may be additional reservoirs of CDI not currently being highlighted for infection prevention and antimicrobial stewardship interventions.

Rates of HA-CDI can be used to benchmark a healthcare facility’s performance [12]. However, the defining 48-hour admission rule used for HA-CDI origin is not reflective of the fact that the suggested median CDI incubation period lies between 1 and 20 days, which may lead to the over estimation of HA-CDI [13,14]. This is a particular issue for countries where HA-CDI rates and target setting may result in financial penalties to drive reductions in CDI rates IPC [15].

In this study, we wanted to investigate if changing current CDI surveillance definitions to better reflect healthcare utilisation and to account for a more realistic incubation period for *C. difficile* might change classifications of patients according to CDI origin, with potential implications for IPC and antimicrobial stewardship measures.

## Methods

We reviewed new CDI cases between 2012 and 2021 in a large tertiary centre in Ireland and firstly described our CA-CDI patients. We then quantified the impact of altering CDI surveillance definitions in place to better reflect healthcare utilisation and to account for the actual CDI incubation period, which is often greater than 48 hours.

### Study design and period

We conducted a retrospective observational cohort study investigating new (first episode) CDI data from patients in our institution between 1 January 2012 and 31 December 2021. *C. difficile* results are prospectively captured in a secure centralised CDI database with weekly interdisciplinary assignment of CDI case type, origin (HA-CDI, CA-CDI, RD-CDI), date of onset of symptoms and disease severity, as per national case definitions [4,16]. Case type refers to new or recurrent CDI, as per the European Centre for Disease Prevention and Control (ECDC) case definitions, whereas severity is a subset of each (new or recurrent) with a more severe infection, also defined by the ECDC case definitions [4]. The first new episode of CDI per patient by origin was identified and included in the analysis.

### Setting, *Clostridioides difficile *testing and infection management

Beaumont Hospital, Dublin, is an adult (i.e. patients aged 18 years and older) tertiary referral centre with over 800 beds. The onsite microbiology laboratory performs daily *C. difficile* testing using a two-step testing protocol on all stool samples which take the shape of the stool container, irrespective of clinician request [11]. As part of routines, positive results and their clinical significance are communicated daily to the primary clinical team by the clinical microbiologist, with CDI management recommendations, as appropriate.

### Cases and collected data

Patient details with positive *C. difficile* laboratory results are captured prospectively in a centralised CDI database, which collects information on patient demographics (age, sex, length of hospitalisation), patient location at time of symptom onset, CDI case type and origin, patient outcome at discharge and RT. Data on day attendances are not included routinely in this surveillance. The CDI database is quality-assured monthly by a consultant microbiologist and a surveillance scientist.

### Demographic differences between admitted and non-admitted patients with new community-associated *Clostridioides difficile* infections (CA-CDI)

The demographics of new CA-CDI patients, both those admitted and not admitted to hospital, were investigated and patients’ files reviewed for any hospital attendances including those without an overnight stay in the 12 weeks before CDI. Differences between the admitted and non-admitted CA-CDI patients were investigated using ꭓ^2^ for categorical and Wilcoxon rank sum test for continuous variables. All analyses were performed using Stata version 16.1. Associations with a p < 0.05 were considered significant.

### Attendance data collected and assignment criteria for healthcare-exposure *Clostridioides difficile* infection (HE-CDI)

Using the patient administration system, the number of attendances and attendance category within the preceding 12 weeks of CDI detection were recorded. A day case was defined as an admission to the hospital for care or treatment without an overnight stay. Attendance was categorised into six categories: day case, emergency department (no inpatient admission), oncology day ward, haematology day ward, haemodialysis or radiology appointments. Any patients with oncology, haematology, day or haemodialysis attendances were reassigned as healthcare-exposure (HE)-CDI. Patients attending the emergency (without admission) and radiology departments were only included as HE-CDI if they had more than two attendances in the 12 weeks prior to CDI, as per current case definitions [3,4]. RD-CDI patients were also reassigned as HE-CDI as described in the Supplementary Material.

### Sensitivity and specificity analysis of onset time for assigning healthcare-associated *Clostridioides difficile* infection (HA-CDI)

To investigate the time used to distinguish between CA-CDI and HA-CDI, the time duration required from admission to onset when assigning ‘HA-CDI’ was varied between 1 and 10 days, as this better reflects the known incubation period of CDI [13,14]. Two possible cut-off points were reviewed. The first was to balance sensitivity and specificity where the levels of each were similar (‘balanced cut-off’). The second, balanced sensitivity with the positive predictive value (‘optimal cut-off’) [17]. The midpoint between optimal and balanced cut-off, rounded to the nearest whole number, was used to inform a new cut-off to assign HA-CDI. The new cut-off identified was used (rather than 48 hours) to redefine to HA-CDI and numbers of patients fitting in this redefined category were compared to numbers in the initial HA-CDI category.

### Comparison of case distribution depending on definitions used

Finally, we applied the new HE criteria and changed the cut-off time to define categories in the existing database, and compared the resulting distribution of CA-CDI, HA-CDI and HE-CDI with the original distribution of CA-CDI, HA-CDI, and RD-CDI.

## Results

Of 1,047 patients with new-onset CDI, 801 (77%) were HA-CDI, 205 (20%) CA-CDI and 41 (4%) were RD-CDI using the current definition. Table 1 summarises the CA-CDI patient cohort over the 10 years. Most CA-CDI (n = 140; 68%) were female, and half (n = 103; 50%) were over 65 years of age. Compared with HA-CDI patients, CA-CDI patients were significantly younger (50% under 65 years vs 30% (236/801) of HA-CDI; p < 0.01), with more female patients (68% vs 54% (432/801) HA-CDI; p < 0.01). The median length of stay (LoS) of CA-CDI patients hospitalised for any reason was 9 days, similar to RD-CDI (10.5 days) but significantly different from HA-CDI patients (LoS of 31 days; p < 0.01). Of the 168 admitted CA-CDI patients, 135 (80%) were discharged home, 22 (13%) transferred to another healthcare facility and 11 (7%) died during their admission. 

**Table 1:** Characteristics of new community-associated *Clostridioides difficile* infections (CA-CDI), overall and by admission or not to hospital, Beaumont Hospital, Dublin, Ireland, 1 January 2012−31 December 2021 (n = 205)

Characteristic	Total CA-CDI			Hospital admission			No hospital admission		
	Number	Total	%	Number	Total	%	Number	Total	%
**Sex**									
Male	65	205	31.7	46	168	27.4	19	37	51.4
Female	140		68.3	122		72.6	18		48.6
**Age**									
≤ 29 years	22	205	10.7	13	168	7.7	9	37	24.3
30 to 49 years	41		20.0	32		19.1	9		24.3
50 to 64 years	39		19.0	34		20.2	5		13.5
≥ 65 years	103		50.3	89		53.0	14		37.8
**Sample request type**									
Clinician CDI test request	28	205	13.7	24	168	14.2	4	37	10.8
Faecal specimens tested (no CDI request)	177		86.3	144		85.7	33		89.2
**Source of initial diagnostic sample**									
Emergency department	15	205	7.3	1	168	0.6	14	37	37.8
Hospital in-patient ward	157		76.6	157		93.5	0		0.0
GP sample	5		2.4	0		0.0	5		13.5
Day ward	13		6.3	0		0.0	13		35.1
Renal day ward	15		7.3	10		6.0	5		13.5
Severe CDI^a^									
Severe CDI^a^	3	205	1.5	3	168	1.8	0	37	0.0
**Events in the 12 weeks prior to CDI onset**									
Hospital day service attendance	75	205	36.5	59	168	35.1	16	37	43.2
Hospitalisation (or not) among 75 prior hospital-day-service attenders	NA	NA	NA	59	75	78.7	16	75	21.3
Antimicrobials in the 12 weeks before CDI onset^b^	81	131^b^	61.8	72	107^b^	67.3	9	24^b^	37.5
**CDI treatment^b^**									
Fidaxomicin	66	131^b^	50.4	56	107^b^	52.3	10	24^b^	41.7
Metronidazole	29		22.1	18		16.8	11		45.8
Vancomycin	25		19.1	25		23.4	0		0.0
Not available	11		8.4	8		7.5	3		12.5

CDI: *Clostridioides difficile* infection; GP: general practioner; NA: not applicable.

^a^ Severe CDI is defined as a CDI patient to whom any of the following criteria apply: (i) admission to an intensive care unit for treatment of CDI or its complications (e.g. for shock requiring vasopressor therapy); (ii) surgery (colectomy) for toxic megacolon, perforation, or refractory colitis [3,4].

^b^ Available data on antimicrobial exposure before CDI onset and details of CDI treatment were only captured from 2016.

Seventy-five (37%) of the 205 CA-CDI patients had attended the hospital without an overnight admission in the 12 weeks prior to CDI (Table 2). In this period, the overall number of day attendances from this 75-patient group was 348 day attendances. Within the 75-patient group, the number of haemodialysis patients represented 5%, however, the attendances of such patients accounted for 41% of the total day attendances. While haematology patients represented 7% of the group, they accounted for 21% of the total day attendances with a median of 12 day attendances per patient (interquartile range: 11–21). Forty-three patients had attended day, haematology, oncology or haemodialysis wards. A further two patients were identified as having had two or more emergency or radiology attendances in the 12 weeks before CDI onset and met the HE criteria. Overall, 60% (n = 45) of the 75 patients with CA-CDI met the HE criteria. When the HE criteria (Supplementary Material) were applied 45/205 (22%) CA-CDI patients were reassigned as HE-CDI, which resulted in 15% CA-CDI, 8% HE-CDI and 77% HA-CDI (Table 3).

**Table 2:** Distribution, according to day attendance categories, of CA-CDI patients who were not admitted overnight in the 12 weeks before CDI diagnosis, with the median and IQR of number of day attendances by category type, Beaumont Hospital, Dublin, Ireland, 1 January 2012–31 December 2021 (n = 75)

Category number	Day attendance category	Patients with hospital attendance without overnight admission		Attendances of the patients without overnight admission			
		Number of patients	%	Number of day attendances	%	Median	IQR
1	Day ward	30	40	64	18	1	1–3
2	Haematology day services	5	7	74	21	12	11–21
3	Oncology day services	4	5	22	6	4	3–8
4	Haemodialysis treatment	4	5	144^a^	41	NA	NA
5	Emergency department^b^	24	32	35	10	1	1–2
6	Radiology^b^	8	11	9	3	1	1–1
**Total**		**75**	NA	**348**	NA	NA	NA

CA-CDI: community acquired *Clostridioides difficile* infection; CDI: *Clostridioides difficile* infection; HE: healthcare exposure; IQR: interquartile rage; NA: not applicable.

^a^ Each patient attended for dialysis three times weekly in the 12 weeks prior to CDI onset.

^b^ Only patients with two or more attendances in the 12 weeks before CDI diagnosis were included in the HE category.

**Table 3:** Number of *Clostridioides difficile* infection (CDI) by origin using initial and revised surveillance definitions to include an incubation period for CDI of 4 days and patients with recent healthcare exposure, Dublin, Ireland, 1 January 2012–31 December 2021 (n = 1,047)

Revision		CA-CDI	RD/unknown-CDI	HA-CDI	Revised total (N = 1,047)	
					Number	%
None (current definition [3,4])		205	41	801	NA	NA
HE origin added	CA	160	0	0	160	15
	HE	45	41	0	86	8
	HA	0	0	801	801	77
4-day cut-off used to assign HA-CDI	CA	205	0	46	251	24
	RD	0	41	0	41	4
	HA	0	0	755	755	72
HE origin added and 4 days cut-off used to assign HA-CDI	CA	160	0	46	206	20
	HE	45	41	0	86	8
	HA	0	0	755	755	72

CA: community-associated; CDI: *Clostridioides difficile* infection; HA: healthcare associated; HE: healthcare exposure; NA: not applicable; RD: recent discharge.

The Figure summarises the sensitivity and specificity analysis of varying the cut-off time parameters (hospital admission to CDI onset). The balanced cut-off for assigning HA-CDI, where sensitivity and specificity were similar, was day 4.5 after admission. The optimal cut-off, where sensitivity and positive predictive values were similar for assigning HA-CDI, was day 3 onwards. Day 4 was selected as the new cut-off time point, as it represented a mid-way between the two cut-offs, while also being nearest a whole number. When the day 4 cut-off was applied, 46/801 (6%) HA-CDI patients were reassigned as CA-CDI, which resulted in 24% CA-CDI, 4% HE-CDI and 72% HA-CDI (Table 3).

**Figure:**
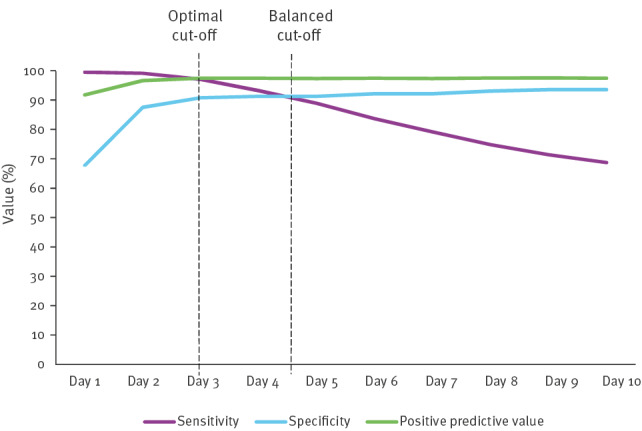
Hospital associated *Clostridioides difficile* infection (CDI) cut-off limits between 1 and 10 days with associated sensitivity, specificity and positive predictive value using data from patients with new CDI admitted to Beaumont Hospital, Dublin, Ireland, 1 January 2012−31 December 2021 (n = 986 CDI patients admitted)

Optimal cut-off: similar sensitivity and positive predictive value.

Balanced cut-off: similar sensitivity and specificity.

Lastly, we applied both the new HE-CDI origin and new 4-day cut-off to our original dataset. This led to 45 CA-CDI patients being re-categorised as HE-CDI and 46 HA-CDI as CA-CDI. The overall distribution was then calculated to be 20% CA-CDI, 8% HE-CDI and 72% HA-CDI (Table 3). The characteristics of the revised CA cohort remained similar to that of the original CA: 67% (140/206) female, 53% (111/206) of cases aged over 65 years. However, the median LoS among the 179 admitted increased from 9 days to 17 days.

## Discussion

Overall, we found that varying the parameters of CDI surveillance definitions applied to our patient cohort data did not hugely skew distributions of CDI by origin but resulted in a classification of CDI origin more reflective of the pathogen’s characteristics and of patient’s exposure risk. In the original distribution: 76% were HA-CDI, 20% CA-CDI and 4% were RD-CDI. In the revised distribution, 72% were HA-CDI, 20% CA-CDI, and 8% were categorised as HE-CDI (previously RD). Increasing the cut-off time to assign HA-CDI cases from 48 hours after hospital admission to 4 days, reflects the reported CDI incubation period and reduces potential over-estimates of HA-CDI, which in our case was by 6%. By modifying the RD-CDI origin category to a broader category encompassing HE-CDI origin, we identified a subset of ‘CA-CDI’ patients with hospital day attendances that we could monitor and track for IPC and antimicrobial stewardship measures.

Surveillance for CDI requires standardised case definitions to benchmark and monitor the impact of IPC and antimicrobial stewardship programmes. At national level, these data can also be used to inform healthcare policy and services and help direct where resources are best deployed. It is recommended for epidemiological surveillance systems that their sensitivity, specificity and representativeness be regularly considered [5,18]. Healthcare utilisation has changed significantly over the last decade with reductions in LoS and patients increasingly accessing hospital day services, without being admitted to hospital [11,19,20]. In addition, the 48-hour time cut-off to assign HA-CDI, while consistent with other healthcare-associated infection surveillance programmes is not reflective of the characteristics of the pathogen. We believe that the current approach to CDI surveillance has not adapted to reflect these considerations.

Identification of CDI in the hospital day settings has been noted as a gap in current surveillance definitions [21-23]. In Ireland, the national protocol has been recently updated to include ‘ambulatory care’ patients in the outpatient setting. However, these patients are still classified as CA-CDI [24]. In the study reported here, one in five patients originally assigned as CA-CDI had two or more recent hospital contacts before developing CDI, which likely impacted their CDI risk. CDI surveillance systems need to accurately capture such patients, hence, the proposal to include them in an adapted HE-CDI origin.

The HE-CDI origin would also capture patients who develop clinical disease after hospital discharge, which is of particular importance as hospital LoS decreases. Shorter LoS present challenges for post-discharge surveillance programmes, as it can be more difficult to capture accurately and categorise these patients as current definitions underestimate the true extent of HA-CDI. Likewise, the inability to specifically identify CDI in the hospital day setting presents a missed opportunity for infection prevention and antimicrobial stewardship. Our current CDI prevention programmes are focused primarily on interventions for hospitalised patients and emergency department attendees. There is less focus on patients who attend the hospital for day services. By increasing awareness of CDI, its symptoms and encouraging testing in the day care setting, timely CDI diagnosis and management is facilitated, with associated benefits for patients and hospital budgets. In addition, it would improve our understanding of *C. difficile* transmission across healthcare and home settings. By including the ‘recently discharged’ origin with hospital attendances in the proposed HE-CDI origin, it provides a surveillance system that is more accurate and reflective of the CDI risk from increased healthcare exposure. The IPC and antimicrobial stewardship governance within these clinical areas are distinct and merit their own focus from an improvement perspective.

Further consideration of the cut-off time-point for assigning HA-CDI is also required. The current 48-hour exposure window is not reflective of *C. difficile* as a pathogen and could be overestimating the true incidence of HA-CDI, in our hospital by as much as 6%. This may inadvertently misdirect IPC efforts into investigating CDIs that are more likely to be CA-CDI and is especially important in those healthcare systems that impose financial penalties when HA-CDI targets are breached [25]. The reported incubation period for *C. difficile* varies greatly [17,26]. Using a statistical approach by conducting sensitivity and specificity analysis on our 10-year dataset to approximate a cut-off point for assigning HA and CA-CDI, we have identified a day 4 cut off point. This estimation is based on a theoretical framework, which could possibly be representative of CDI compared to applying a 48-hour cut-off.

Increased focus on benchmarking of healthcare facilities according to rates of HA-CDI, underscores the need for more plausible CDI definitions. A 2018 study reported that the 2-day cut-off overestimates HA-CDI and suggested using either a 5 or 6-day cut-off for the HA-CDI origin, while another study supported a day 5 cut-off [17,26]. The sensitivity analysis conducted in our study suggests a day 4 cut-off but is limited to a single site. Further work in other centres would be helpful when examining the most suitable cut-off for the wider population.

Limitations of this study include it being a single centre study, and information on hospital day attendances in healthcare facilities other than Beaumont Hospital was not available. However, the strengths of this study are the robustness of analysed data with prospective standardised CDI case categorisation and validation by the same multidisciplinary team, monthly quality assurance of the CDI database and RT performed on all positive samples by the same laboratory.

### Conclusion

CDI surveillance definitions have provided a standardised framework that is used widely to protect patients and inform policy. In light of changing healthcare utilisation and the knowledge that an incubation period of 48 hours (2 days) likely does not reflect the epidemiology of CDI (even if it may do so for other healthcare-associated infections), we believe that it is time to discuss and assess changes to the current CDI surveillance definitions. This might start with other centres applying our alterations and considering possible other changes to the definitions, backed up by accurate diagnostic methods, and consistent data collection. This would provide a better understanding of the epidemiology of CDI in Europe and beyond, better inform policy, and help best protect at-risk patients.

## Note

The study followed the Strengthening the Reporting of Observational studies in Epidemiology (STROBE) guidelines [27].

## Supplementary Data

127000 Supplementary Material
