# Supplementary material for: Rethinking Clostridioides difficile infection (CDI) surveillance definitions based on changing healthcare utilisation and a more realistic incubation period: reviewing data from a tertiary-referral hospital, Ireland, 2012 to 2021
Source: Euro Surveill. 2024 Feb 8;29(6):2300335. doi: 10.2807/1560-7917.ES.2024.29.6.2300335 (PMC10853979; doi:10.2807/1560-7917.ES.2024.29.6.2300335)
Supplement: Supplementary Material [file 2300335_SupplementaryMaterial.pdf]

This supplementary material is hosted by *Eurosurveillance* as supporting information alongside the article 'Rethinking *Clostridioides difficile* infection (CDI) surveillance definitions based on changing healthcare utilisation and a more realistic incubation period: reviewing data from a tertiary-referral hospital Ireland, 2012 to 2021', on behalf of the authors, who remain responsible for the accuracy and appropriateness of the content. The same standards for ethics, copyright, attributions and permissions as for the article apply. Supplements are not edited by *Eurosurveillance* and the journal is not responsible for the maintenance of any links or email addresses provided therein.

Supplementary Material: Current Origin of CDI Definitions and Altered Origin of CDI

Origin of CDI category	Current definition [3, 4]
**Community associated (CA)**	Onset of symptoms while outside a healthcare facility and without discharge from a healthcare facility within the previous 12 weeks.<br>Onset of symptoms within 48 hours following admission to a healthcare facility without residence in a healthcare facility within the previous 12 weeks.
**Healthcare associated (HA)**	Onset of symptoms at least 48 hours following admission to a healthcare facility.<br>Onset of symptoms in the community within four weeks following discharge from a healthcare facility.
**Discharged 4-12 weeks from healthcare facility (RD) / Unkown**	Patient was discharged from a healthcare facility between four and 12 weeks before the onset of symptoms.

Origin of CDI category	Altered definition
**Community associated (CA)**	Onset of symptoms while outside a healthcare facility and without discharge from a healthcare facility within the previous 12 weeks.

	Patient did not have any day case, oncology day ward, haematology day ward, haemodialysis attendances or had less than two radiology or emergency department attendances within the previous 12 weeks.<br>Onset of symptoms within 48 hours following admission to a healthcare facility without residence in a healthcare facility within the previous 12 weeks.
**Healthcare associated (HA)**	Onset of symptoms at least 4 days following admission to a healthcare facility (healthcare-onset, healthcare-associated).<br>Onset of symptoms in the community within four weeks following discharge from a healthcare facility (community-onset, healthcare-associated).
**Healthcare exposure (HE)**	Patient was discharged from a healthcare facility between four and 12 weeks before the onset of symptoms.<br>Patient had one or more day case, oncology day ward, haematology day ward or haemodialysis attendances or two or more radiology or emergency department attendances within the previous 12 weeks.
